# Reliability, validity and discriminant ability of a robotic device for finger training in patients with subacute stroke

**DOI:** 10.1186/s12984-019-0634-5

**Published:** 2020-01-03

**Authors:** Marco Germanotta, Valerio Gower, Dionysia Papadopoulou, Arianna Cruciani, Cristiano Pecchioli, Rita Mosca, Gabriele Speranza, Catuscia Falsini, Francesca Cecchi, Federica Vannetti, Angelo Montesano, Silvia Galeri, Furio Gramatica, Irene Aprile, the FDG Robotic Rehabilitation Group

**Affiliations:** 1 IRCCS Fondazione Don Carlo Gnocchi, Via di Scandicci, 269, 50143 Florence, Italy; 2 IRCCS Fondazione Don Carlo Gnocchi, Piazzale Morandi 6, 20121 Milan, Italy

**Keywords:** Stroke, Hand, Rehabilitation, Upper extremity, Robotics, Reliability, Validity, Discriminant ability

## Abstract

**Background:**

The majority of stroke survivors experiences significant hand impairments, as weakness and spasticity, with a severe impact on the activity of daily living. To objectively evaluate hand deficits, quantitative measures are needed. The aim of this study is to assess the reliability, the validity and the discriminant ability of the instrumental measures provided by a robotic device for hand rehabilitation, in a sample of patients with subacute stroke.

**Material and methods:**

In this study, 120 patients with stroke and 40 controls were enrolled. Clinical evaluation included finger flexion and extension strength (using the Medical Research Council, MRC), finger spasticity (using the Modified Ashworth Scale, MAS) and motor control and dexterity during ADL performance (by means of the Frenchay Arm Test, FAT). Robotic evaluations included finger flexion and extension strength, muscle tone at rest, and instrumented MAS and Modified Tardieu Scale. Subjects were evaluated twice, one day apart, to assess the test-retest reliability of the robotic measures, using the Intraclass Correlation Coefficient (ICC). To estimate the response stability, the standard errors of measurement and the minimum detectable change (MDC) were also calculated. Validity was assessed by analyzing the correlations between the robotic metrics and the clinical scales, using the Spearman’s Correlation Coefficient (r). Finally, we investigated the ability of the robotic measures to distinguish between patients with stroke and healthy subjects, by means of Mann-Whitney U tests.

**Results:**

All the investigated measures were able to discriminate patients with stroke from healthy subjects (*p* < 0.001). Test-retest reliability was found to be excellent for finger strength (in both flexion and extension) and muscle tone, with ICCs higher than 0.9. MDCs were equal to 10.6 N for finger flexion, 3.4 N for finger extension, and 14.3 N for muscle tone. Conversely, test-retest reliability of the spasticity measures was poor. Finally, finger strength (in both flexion and extension) was correlated with the clinical scales (r of about 0.7 with MRC, and about 0.5 with FAT).

**Discussion:**

Finger strength (in both flexion and extension) and muscle tone, as provided by a robotic device for hand rehabilitation, are reliable and sensitive measures. Moreover, finger strength is strongly correlated with clinical scales. Changes higher than the obtained MDC in these robotic measures could be considered as clinically relevant and used to assess the effect of a rehabilitation treatment in patients with subacute stroke.

## Background

After stroke, most of the patients experiences a deficit at the hand and, six months after the acute event, about 65% of patients cannot incorporate the affected hand into their usual activities [1]. The ability to perform activities of daily living (ADL) is highly dependent on hand function, leaving those suffering with hand impairments less capable of executing ADL and consequently with a reduced quality of life [2]. Grip, strength, and overall functions of the hands are often impaired, making everyday tasks hard to accomplish and consequently compromising severely the ability to be independent in functional activities. In severe patients, the injured hand very often remains plegic, with difficulty extending the fingers [3] and no marked recovery over time [4, 5], making the recovery of hand function one of the most challenging topics in stroke rehabilitation [6].

Rehabilitation of arm function after stroke has been changing substantially over the last decades [7] but, up to now, the optimal intervention is far from being identified. In the last decades, a growing interest has been addressed towards the use of robotic devices to treat the upper limb in patients suffering from neurological disease, especially stroke [8]. In fact, these devices allow to increase of the amount and intensity of the therapy, to standardize the treatment, providing a complex but controlled multisensory stimulation [9, 10] and helping the patient to complete the required task while preventing inappropriate movements [11]. Even if most of the robots focuses on the more proximal joints (shoulder and elbow) [12], some devices have been specifically developed to target the hand, using either end-effector [13–15] or exoskeleton [16, 17] design, with encouraging results in terms of motor recovery [12, 18–22].

In addition, robotic devices, because of their built-in technology in terms of sensors and actuators, are able to objectively quantify the motor status of patients after brain damage, as well as their motor recovery. In fact, such devices are able to acquire kinematic and kinetic data which are processed to obtain quantitative indices [23–34]. These robot-derived measures can potentially add meaningful information about the patient’s performance, helping the clinicians in patient’s assessment. As a condition of their use in clinical practice, however, their properties in terms of reliability, validity and responsiveness should be assessed. In fact, in order to be brought into the clinical field, the obtained measures have to be stable, sensitive and clinically meaningful.

Amadeo (Tyromotion, Austria) is a mechatronic end-effector robotic device specifically designed to treat the hand. Results from its application in stroke patients suggest its efficacy in reducing hand impairment [20, 22, 35, 36]. To the best of our knowledge, however, the psychometric properties of the measures provided by this robotic device have not yet been investigated. Therefore, the aim of the present work is to evaluate, within a multicenter randomized controlled trial, the reliability, the concurrent validity and the discriminant ability of the indices provided by a robotic rehabilitation device for hand rehabilitation.

## Materials and methods

### Participants

In this study, we analyzed the data obtained from 120 consecutive patients with subacute stroke, enrolled in 6 different rehabilitation centers of the Fondazione Don Carlo Gnocchi (Rome, Milan, Florence, Sant’Angelo dei Lombardi, Rovato and Fivizzano) This is a cross-sectional analysis of baseline data collected as part of a larger clinical trial [37], approved by the institutional ethics committee (FDG_6.4.2016) and registered at clinicaltrials.gov with identifier number NCT02879279.

Inclusion criteria were: (1) first-ever stroke (cerebral infarction or hemorrhage), confirmed by either brain CT or MRI findings (2) age between 40 and 85 years; (3) time since stroke onset less or equal to 6 months; (4) cognitive and language abilities sufficient to understand the experiments and follow instructions. Exclusion criteria were: (1) upper extremity Fugl-Meyer score > 58; (2) behavioral and cognitive disorders and/or reduced compliance that would interfere with active therapy; (3) fixed contraction deformity in the affected limb that would interfere with active therapy (ankylosis, Modified Ashworth Scale = 4); (4) inability to discriminate distinctly the images showed on a monitor placed at the eye level of each subject at a distance of about 50 cm, even with corrective glasses. Demographic and characteristics of the patients are shown in Table 1. In addition, 40 age and sex matched subjects without neurological or other relevant medical conditions served as a reference population. All participants gave their written informed consent according to the Declaration of Helsinki.

**Table 1:** Demographic and clinical characteristics of the sample

	Patients with stroke (*N* = 120)
Age (years)	69.4 (10.7)
Sex (M/F)	68/52
Ischemic/Hemorragic	95/25
Time since stroke (days)	48.2 (43.7)
Fugl-Meyer Assessment - Upper Extremity	24.3 (16.5)
*Proximal*	11.7 (8.2)
*Wrist/hand*	9.2 (8.1)
*Coordination/speed*	3.4 (1.3)
MRC finger extension	1.6 (1.6)
MRC finger flexion	1.4 (1.6)
Frenchay Arm Test	1.1 (1.8)
MAS (fingers)	0.4 (0.8)

Data are mean (SD), or numbers

### Clinical assessment

Patients were clinically evaluated using the Medical Research Council (MRC) [38], the Modified Ashworth Scale (MAS) [39] and the Frenchay Arm test (FAT) [40]. The MRC is an ordinal scale for muscle power, ranging from 0 to 5 in relation to the maximum expected for that muscle; in the present work, we evaluated finger flexor and finger extensor. The MAS is an ordinal scale used for grading the resistance encountered during passive muscle stretching, ranging from 0 (normal muscle tone) up to 4 (limb rigid in flexion or extension). The FAT is a measure of upper extremity proximal motor control and dexterity during ADL performance in patients with impairments resulting from neurological conditions, ranging from 0 (worse) to 5 (best). Clinical scales were selected according to a published protocol for upper limb robotic rehabilitation [41].

### Equipment

**Fig. 1:**
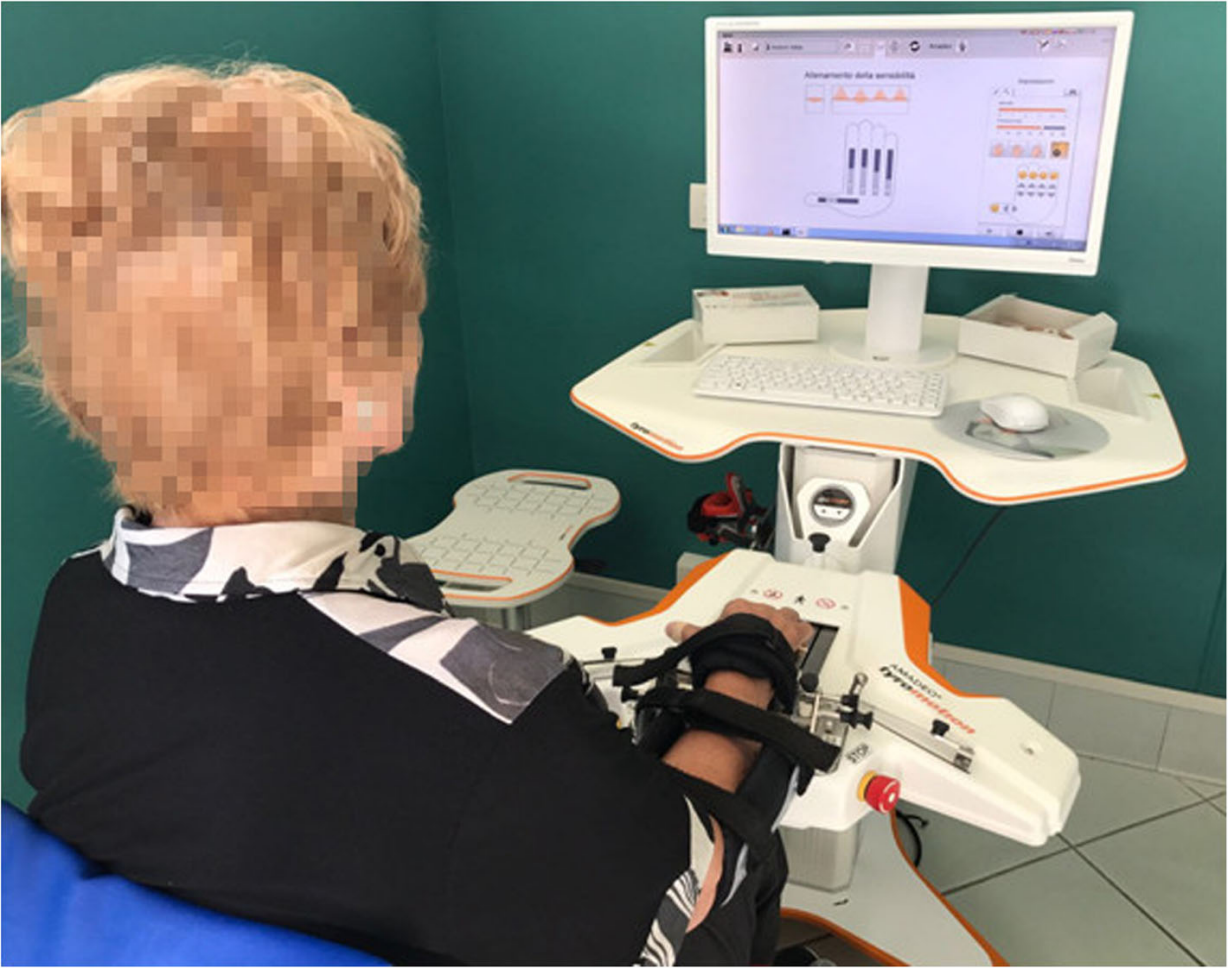
The Amadeo device (Tyromotion)

Amadeo (Tyromotion, Austria) is a robot specifically design for hand rehabilitation (see Fig. 1). It is is an end-effector robot, with 5 degrees of freedom (DOF). It provides the motion of one or all five fingers, thanks to a passive rotational joint placed between fingertip and an entity moving laterally (the thumb has got two passive rotational joints). All five translational DOFs are independent and provide large coverage of the finger workspace. The set-up involved securing a small magnetic disc to the pulp of each finger with adhesive tape for connection with the end-effector, which would move back and forth within sliders aligned with the finger movement direction. The wrist is immobilized using a Velcro strap so that the elbow and shoulder are inhibited from moving. The robot can calibrate the full passive range of motion (pROM) for each finger before the start of a session, and supply the assistive force to patients to complete the remaining range of motion during an exercise. Moreover, the maximum flexion and extension force for each finger are recorded to calibrate the exercise when a strength control is required [36].

### Robotic assessment

**Fig. 2:**
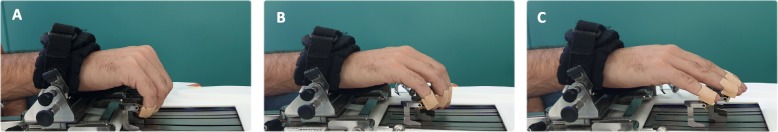
Different positions of the fingers during the tests: maximum flexion (**a**), middle position (**b**) and maximum extension(**c**)

By means of Amadeo, three different evaluations were carried out: force assessment, muscle tone assessment and spasticity assessment. Before starting the assessment, the pROM for each finger and the thumb are recorded for each individual subject (Fig. 2 a-c).

#### Force assessment

The force assessment program measures the patient’s isometric strength for each finger separately and the hand strength. First, the sliders independently move the fingers to the middle position (50% of the pROM, Fig. 2 b). Then, the subject is asked to flex and then to extend his/her fingers as much as possible, while the sliders are blocked. The device measures continuously the force exerted, in both directions by each finger. Finally, the device provides, for flexion and extension separately: the maximum force values independently achieved by each finger; (b) the maximum value of the hand force (obtained as sum of the forces simultaneously exerted by each finger). The latter were referred as HandForce_flex_ and HandForce_ext_ and investigated in the present study. During the test, the physical therapist stimulated the subject to flex and then to extend the fingers as much as possible.

#### Muscle tone assessment

The muscle tone assessment measures the passive baseline force of the finger muscles. The finger sliders move to the middle position (Fig. 2 b). After starting the measurement, the patient tries to keep the fingers as still and relaxed as possible, while the device measures the force exerted by each finger. The measurement lasts 5 s. Before the test, the physical therapist stimulated the subject to relax his/her fingers, while no specific feedback was given during the test.

#### Spasticity assessment

For the spasticity assessment, the fingers can be moved with three varying speeds, which allows a spasticity evaluation based on the Modified Ashworth Scale (MAS) and the Modified Tardieu Scale (MTS). The finger sliders move each finger to the respective starting position, as measured during the baseline pROM assessment. Then, fingers are moved across the entire pROM with three different velocities: V1 (slow), V2 (medium) and V3 (fast). Velocities are set in a way, that all fingers start and arrive at the same time (i.e. the finger with the shortest ROM moves slower than the finger with the largest ROM). V1, V2, and V3 related to the finger with the biggest ROM as follows: V1 = 0.01 m/s; V2 = 0.05 m/s; and V3 = 0.1 m/s. Before the test, the physical therapist stimulated the subject to relax his/her fingers, while no specific feedback was given during the test. According to the clinical measures of spasticity (MAS and Tardieu Scale), the following values are provided: MAS (one value for each velocity); MTS (one value for each velocity); R2 (the full range of motion, calculated at V1, and expressed as percentage of the pROM); R1 (the angle of muscle reaction to the stretch, calculated at V2 and V3, and expressed as percentage of the pROM); the difference R2-R1; a single evaluation for each finger, as well as a total evaluation for the four fingers are provided. In the current study, the MAS and the MTS values, at V1 and V3, were investigated (namely MAS_V1_, MAS_V3_, MTS_V1_, MTS_V3_).

### Experimental protocol

In our study, each participant was asked to perform each investigated assessment provided by the device three times. Specifically, to avoid the onset of spasticity due to the maximum contractions, the order of the tests was the following: 1) muscle tone (three repetitions); 2) spasticity (three repetitions); 3) strength (three repetitions). For each subject, a session lasted between 5 and 10 min, depending on patient’s impairment and compliance. In each rehabilitation center, the robotic assessment was performed by a single physical therapist, proficient in the use of the device. Before starting the study, the procedures were harmonized among centers.

Both patients and healthy subjects were tested twice, 1 day apart, to assess the test-retest reliability of the provided outcome measures. Each subject was evaluated in the two sessions by the same operator, using the pROM recorded in the first evaluation. For both test sessions, the value of each measure obtained in the three repetitions was recorded. With respect to the numeric data (i.e., HandForce_flex_, HandForce_ext_ and Muscle tone), the mean value was computed and used for the statistical analysis. With respect to the ordinal data (MAS_V1_, MAS_V3_, MTS_V1_, MTS_V3_), the best value (i.e., the lowest value) was used.

### Statistical analysis

#### Test-retest reliability

Test-retest reliability of the numeric data was assessed by using the Intraclass Correlation Coefficient (ICC), using a two-way mixed effect, absolute agreement, multiple measurements model. Reliability was classified as excellent (ICC > 0.90), good (0.75 < ICC ≤ 0.90), moderate (0.5 < ICC ≤ 0.75) or poor otherwise [42]. Absolute test-retest reliability was analyzed comparing for each index data obtained during the two test sessions by mean of Wilcoxon signed-rank test and Bland-Altman plots. To estimate the response stability, standard errors of measurement (SEM) and minimum detectable changes (MDC) were also calculated. The SEM were calculated using the mean of the standard deviations (SD) of data obtained at the two paired sessions and the ICC with the following formula:

1 $$ SEM={SD}_{mean}\times \sqrt{1- ICC} $$

while the MDC values were computed using the following formula:

2 $$ MDC=1.96\times SEM\times \sqrt{2}. $$

#### Concurrent validity

To assess the concurrent validity of the robotic indices, the correlations between the robotic parameters and the clinical scales were investigated using the Spearman’s rank correlation coefficients. The coefficient values were interpreted as follows [43]: 0.0–0.2 little if any; 0.2–0.4 weak; 0.4–0.7 moderate; 0.7–1.0 strong.

#### Discriminant ability

The ability of the robotic indices to discriminate stroke patients from healthy subjects was evaluated by means of the Mann-Whitney U test.

Statistical analysis was performed using SPSS (version 25, SPSS Inc., Chicago IL, USA) and MedCalc (version 14, MedCalc Software, Ostend, Belgium). A *p*-value lower than 0.05 was deemed significant. The false discovery rate method [44] was used to adjust for multiple comparisons.

## Results

### Test-retest reliability

**Fig. 3:**
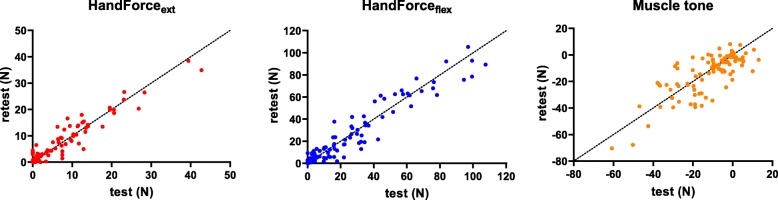
Scatterplot of the three robotic measures showing good reliability

**Fig. 4:**
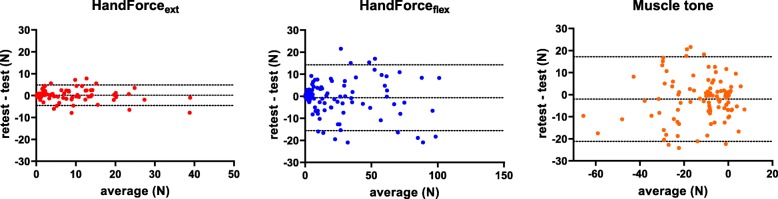
Bland Altman plots of the three robotic measures showing good reliability

ICCs and 95% confidence intervals in patients with stroke, as well as the results of the statistical analysis of the comparison of the two assessments, performed one day apart, are shown in Table 2. Due to clinical reasons, two patients did not perform the retest evaluation. Moreover, due to technical reasons, force and muscle tone data were missing in three and five patients, respectively. Therefore, test-retest reliability was computed using all the available data for each measure (98.3, 95.8 and 94.2% of data for spasticity, strength and muscle tone, respectively). The HandForce_ext_, the HandForce_flex_, and the muscle tone showed an excellent reliability (ICC > 0.9), see Fig. 3, while the MAS and the MTS values showed a poor reliability (ICCs lower than 0.5 for both V1 and V3). With respect to the absolute reliability, only the muscle tone showed a statistical significant increase in the retest, when compared to the test, with a mean increase lower than 2 N. The Bland-Altman plots, with the bias between the two assessments and the limits of agreement for each measure with a good reliability, are reported in Fig. 4.

**Table 2:** Test-retest reliability in patients with stroke

	N	Test Mean (SD)	Retest Mean (SD)	ICC	95% CI		P	SEM	MDC
					Lower bound	Upper bound			
HandForce_ext_ (N)	115	5.7 (8.2)	5.9 (7.8)	0.977	0.967	0.984	0.365	1.2	3.4
HandForce_flex_ (N)	115	22.1 (26.8)	21.5 (26.0)	0.979	0.969	0.985	0.467	3.8	10.6
Muscle tone (N) ^†^	113	−12.0 (16.6)	−13.9 (17.1)	0.906	0.860	0.937	**0.048**	5.2	14.3
MAS_V1_	118	1.2 (1.6)	1.2 (1.5)	0.473	0.240	0.634	0.960	1.1	3.1
MTS_V1_	118	0.8 (1.5)	0.9 (1.5)	0.396	0.129	0.581	0.725	1.2	3.2
MAS_V3_	118	1.6 (1.5)	1.7 (1.5)	0.486	0.257	0.644	0.606	1.1	3.0
MTS_V3_	118	0.9 (1.4)	1.0 (1.5)	0.268	−0.057	0.494	0.424	1.2	3.4

*SD* Standard Deviation; *ICC* Intraclass Correlation Coefficient; *CI* Confidence Interval. *SEM* Standard Error of Measurement; *MDC* Minimal Detectable Change. *P*-values in bold indicate statistical significance of the Wilcoxon signed-rank test (P lower than 0.05). ^†^ Negative values: flexor muscles tone. Positive values: extensor muscles tone

With respect to the healthy subjects (Table 3), a good reliability was found for the HandForce_ext_, the HandForce_flex_, the muscle tone and the MAS_V3_; a moderate reliability for the MAS_V1_; a poor reliability for MTS_V1_ and MTS_V3_. In Table 2 and Table 3 are also reported the SEM and the MDC for all the investigated measures, for stroke patients and healthy subjects, respectively. In stroke patients, MDC values were of 10.6 N for HandForce_flex_, 3.4 N for HandForce_ext_, 14.3 N for muscle tone; for MAS and MTS, MDC values ranged from 3 to 3.4 points.

**Table 3:** Test-retest reliability in healthy subjects (*N* = 40)

	Test Mean (SD)	Retest Mean (SD)	ICC	95% CI		P	SEM	MDC
				Lower bound	Upper bound			
HandForce_ext_ (N)	29.4 (6.7)	29.8 (7.1)	0.735	0.489	0.863	0.338	3.6	9.8
HandForce_flex_ (N)	91.4 (19.5)	92.5 (20.1)	0.888	0.784	0.942	0.388	6.6	18.3
Muscle tone (N) ^†^	−0.2 (0.4)	−0.2 (0.5)	0.839	0.689	0.917	0.161	0.2	0.5
MAS_V1_	0.12 (0.35)	0.31 (0.8)	0.657	0.356	0.819	**0.041**	0.3	0.9
MTS_V1_	0.00 (0.00)	0.10 (0.5)	0.000	−0.880	0.472	0.18	0.5	1.4
MAS_V3_	0.4 (0.7)	0.5 (1.0)	0.849	0.712	0.921	0.474	0.3	1.0
MTS_V3_	0.1 (0.3)	0.2 (0.6)	0.275	−0.362	0.617	0.197	0.4	1.0

*SD* Standard Deviation; *ICC* Intraclass Correlation Coefficient; *CI* Confidence Interval. *SEM* Standard Error of Measurement; *MDC* Minimal Detectable Change. P-values in bold indicate statistical significance of the Wilcoxon signed-rank test (P lower than 0.05). ^†^ Negative values: flexor muscles tone. Positive values: extensor muscles tone

### Concurrent validity

The results of the correlation analysis between the robotic measures and the clinical scales are reported in Table 4. Both the HandForce_flex_ and the HandForce_ext_ showed a strong correlation with the clinical measures of strength (MRC_flex_ and MRC_ext_) and a moderate correlation with the FAT. On the contrary, we did not find a significant correlation neither between muscle tone and the MAS, nor between the robotic and the clinical assessment of spasticity.

**Table 4:** Validity (correlations with clinical scales)

	MRC (ext)	MRC (flex)	FAT	MAS
HandForce_ext_	0.710***	0.732***	0.533***	0.074
HandForce_flex_	0.705***	0.713***	0.550***	0.156
Muscle tone ^†^	−0.05	−0.046	− 0.066	−0.144
MAS_V1_	0.084	0.103	0.042	−0.037
MTS_V1_	0.029	0.059	0.013	0.009
MAS_V3_	0.111	0.091	0.089	0.024
MTS_V3_	0.081	0.064	0.107	−0.040

Correlations between robotic indices and clinical scales are assessed by means of Spearman’s correlation coefficients. *MRC* Medical Research Council; *FAT* Frenchay Arm Test; *MAS* Modified Ashworth Scale. The symbol *** indicates a *P*-value (corrected for multiple comparison, by using a False Discovery Rate procedure) lower than 0.001

### Discriminant ability

The expected ability of the robotic indices to distinguish between patients with stroke and healthy subjects was confirmed by the results of the statistical analysis. In fact, all the robotic indices obtained from patients were statistically different from those of controls (see Table 5), with *p* < 0.001.

**Table 5:** Discriminant ability (differences between patients with stroke and healthy subjects)

	Mean difference (patients - healthy)	SE difference	95% SE		P*
			Lower	Upper	
HandForce_ext_	−23,4	1,5	−26,3	−20,4	**< 0.001**
HandForce_flex_	−68,3	4,8	−77,7	−58,9	**< 0.001**
Muscle tone (N)	−13,8	3,6	−20,9	−6,8	**< 0.001**
MAS_V1_	1,1	0,3	0,6	1,6	**< 0.001**
MTS_V1_	0,8	0,2	0,3	1,3	**< 0.001**
MAS_V3_	1,2	0,3	0,7	1,7	**< 0.001**
MTS_V3_	0,8	0,2	0,3	1,2	**< 0.001**

Comparison between patients with stroke and healthy subjects are assessed by means of the Mann-Whitney U test. *SE* Standard error. Values in bold indicate statistical significance (p lower than 0.05)

^†^The negative value means a higher tone of flexor muscles in patients with stroke, when compared with healthy subjects

* Corrected for multiple comparison, by using a False Discovery Rate procedure

## Discussion

In this study, we assessed the between-day test-retest reliability and the validity of the outcome measures provided by a robotic device for finger training in a sample of patients with subacute stroke, and their ability to differentiate patients from a group of age-matched healthy subjects. The above mentioned outcome measures assess finger strength, both in flexion and in extension, muscle tone at rest, and spasticity measured at different speeds. It is worthy to note that a lower number of studies investigated hand strength, compared to other joint (as shoulder, elbow, knee [45]).

### Strength assessment

Our results showed that both measures of strength (in flexion and in extension), as well as the muscle tone, are characterized by an excellent reliability, with ICC values higher than 0.9, indicating that they could be used for intra-individual comparisons (i.e. for individual decision-making) and not just for group-level comparisons (i.e. for the evaluation of a whole large group of patients), where an ICC value of 0.7 level is acceptable. Moreover, as showed by the statistical analysis, no bias was detected for the measure of strength. Finally, our MDCs, i.e., the minimal change needed to be confident at 95% that the observed change is true, are 10.6 and 3.4 N for flexion and extension force, respectively. This means that a difference in score higher of the above mentioned values can be interpreted as a change in patient’s finger strength, and not due to the measurement error. Our results are in accordance with the previous studies on the same subject. In fact, literature data confirmed that hand strength measures are reliable in patients with stroke. Comparing our results to those already published, obtained with different instrument, we found similar ICCs, and lower (i.e., better) MDCs. Specifically, Bertrand et al. [46], evaluating grip strength in a sample with stroke in the first weeks after a stroke, using a Jamar dynamometer, found ICC ranging from 0.97 to 0.99, and MDC ranging from 2.73 to 4.68 kg. Similarly, Chen et al. [47] investigated the test-retest reproducibility of 3 hand strength tests (grip, palmar pinch, and lateral pinch) both in patients with a recent stroke (onset < 6 months) and in chronic stroke (onset > 6 months), resulting in ICCs ranging from 0.85 to 0.98., while the MDCs for the more/less affected hand were 2.9/4.7 kg for the grip test, 1.2/1.3 kg for the palmar pinch test, and 1.4/1.0 kg for the lateral pinch test. Boissy et al. [48] assessed the maximal voluntary grip force with a modified strain gauge dynamometer in 15 chronic stroke subjects and 10 control subjects, obtaining an ICC > 0.86 and a SEM of 25 N.

Moreover, as expected, patients with stroke showed a strength impairment, both in flexion and in extension. The measures of strength were also well correlated with the corresponding clinical measures of strength, confirming that they provide meaningful information from a clinical point of view. In addition, the correlation with the Frenchay Arm test confirm that the weakness of hand in stroke patients reflects a reduction of upper extremity proximal motor control and dexterity during activity of daily living. The obtained results about the finger strength (high reliability, low MDC, correlation with clinical scales) are very important from a clinical point of view, given the importance of a correct assessment of finger strength, not only to assess the effect of a treatment, either robotic or conventional, but also to tailor the treatment itself, on the basis of the initial status of the patients. In fact, several studies highlighted the importance of finger strength as a predictor of recovery in stroke patients [49–53] and, therefore, to obtain crucial information to manage the rehabilitation pathway.

### Muscle tone at rest

With respect to the muscle tone measurement, we found an excellent reliability, as showed by the ICC value, equal to 0.906. This results is of particular importance, because in patients with upper limb dysfunction following stroke, hypertonicity is a common problem that can contribute to impaired movement patterns and result in significant activity limitations; moreover, usually in the upper limb, flexor muscles are more commonly involved distally [54], supporting the importance of an instrumented assessment for the muscle tone at the hand. In particular, the clinical efficacy of treatments for spasticity would be further improved if the spasticity assessments are more reliable and accurate [55].

In stroke patients, the muscle tone was not correlated with the MAS: however, this result is unsurprising because of (a) the very low variation of the MAS in the analyzed sample (73.1% of patients were clinically rated between 0 and 1); (b) the different assessment, i.e., at rest vs movement, that can lead to different results; and (c) the known low metrological characteristics of the scale itself [56].

### Spasticity

Finally, spasticity measures, i.e. the MAS and the MTS, at both low and high velocity, showed unsatisfactory results from a psychometric point of view. In fact, even if a significant difference with the healthy subjects was found, our results indicate that their reliability was poor, and, in addition, they do not show meaningful correlation with the clinical scale (i.e., the MAS clinically evaluated). With respect to the lack of correlation with the clinical assessment, our results are in accordance with those already reported in literature, where instrumental assessments of spasticity usually appear uncorrelated with the clinical counterparts [57–59]. In fact, the psychometric properties of the clinical assessment of spasticity are very low [60] and, therefore, they cannot act as golden standard, supporting the search of new tools to objectively quantify the spasticity. Conversely, referring to the ICC values, these results differ from some published studies: Centen et al. [61] using a robotic exoskeleton for the upper limb to evaluated the spasticity at elbow, found intra-class correlations that varied, depending on parameter, from 0.66 to 0.95; Condliffe et al. [62], recording biceps brachii and brachioradialis EMG and torque during passive ramp-and-hold elbow flexion, obtained ICC ranging from 0.63 to 0.85. Calota et al. [63], using a portable device, found ICCs from 0.46 to 0.68.

These differences can be explained by several argumentations. First of all, none of the previous studies investigated the finger spasticity; additionally, most of the studies employed both mechanical and neurophysiological measures [60]; moreover, previous studies investigated, as measure of spasticity, mechanical (as torque) or neurophysiological (as EMG burst) measures, not an ordinal measure of spasticity; finally, their cohort comprised stroke patients in the chronic phase, where spasticity is higher and the general conditions of patients are more stable. With respect to the latter aspect, it is worth noting that spasticity, as measured by the MAS, in our sample was low and with low variability across the patients, and this can be considered a limitation of the study. Another limitation is the absence of the evaluation of the responsiveness to treatment of the investigated robotic measures.

## Conclusion

We found that, in a sample of patients with stroke in the subacute phase, the measures of finger flexion and extension strength, provided by a robotic device for finger training are reliable, sensitive and strongly correlated with the clinical scales. Moreover, the measure of muscle tone at rest showed an excellent reliability. Therefore, they can be used as an evaluation tool that can be usefully integrated with the clinical evaluation. Conversely, in the investigated sample, the measures of spasticity did not show similar properties. The instrumental outcome measures are very important to have an objective and easy evaluation, as well as a guide to address the treatment path.

## Data Availability

The dataset used and/or analyzed during the current study available from the corresponding author on reasonable request.

## Abbreviations

ADL: Activities of Daily Living
FAT: Frenchay Arm test
ICC: Intraclass Correlation Coefficient
MAS: Modified Ashworth Scale
MDC: Minimum Detectable Change
MRC: Medical Research Council
MTS: Modified Tardieu Scale
pROM: Full passive Range of Motion
r: Spearman’s correlation coefficient
SEM: Standard Error of Measurement
